# Validation of thoracic aortic dimensions on ECG-triggered SSFP as alternative to contrast-enhanced MRA

**DOI:** 10.1007/s00330-020-06963-x

**Published:** 2020-06-07

**Authors:** G. J. H. Snel, L. M. Hernandez, R. H. J. A. Slart, C. T. Nguyen, D. E. Sosnovik, V. M. van Deursen, R. A. J. O. Dierckx, B. K. Velthuis, R. J. H. Borra, N. H. J. Prakken

**Affiliations:** 1 Department of Radiology, University Medical Center Groningen, University of Groningen, Hanzeplein 1, 9713 GZ Groningen, The Netherlands; 2 Department of Nuclear Medicine and Molecular Imaging, University Medical Center Groningen, University of Groningen, Hanzeplein 1, 9713 GZ Groningen, The Netherlands; 3 Department of Biomedical Photonic Imaging, University of Twente, Dienstweg 1, 7522 ND Enschede, The Netherlands; 4 Department of Radiology, Athinoula A. Martinos Center for Biomedical Imaging, Massachusetts General Hospital, Harvard Medical School, 149 13th Street, Charlestown, MA 02129 USA; 5 Cardiovascular Research Center, Massachusetts General Hospital, Harvard Medical School, 149 13th Street, Charlestown, MA 02129 USA; 6 Division of Health Sciences and Technology, Harvard-MIT, 7 Massachusetts Avenue, Cambridge, MA 02139 USA; 7 Department of Cardiology, University Medical Center Groningen, University of Groningen, Hanzeplein 1, 9713 GZ Groningen, The Netherlands; 8 Department of Radiology, University Medical Center Utrecht, Heidelberglaan 100, 3584 CX Utrecht, The Netherlands

**Keywords:** Thoracic aorta, Magnetic resonance angiography, Reproducibility of results, Contrast media, Cardiac-gated imaging techniques

## Abstract

**Objectives:**

Assessment of thoracic aortic dimensions with non-ECG-triggered contrast-enhanced magnetic resonance angiography (CE-MRA) is accompanied with motion artefacts and requires gadolinium. To avoid both motion artefacts and gadolinium administration, we evaluated the similarity and reproducibility of dimensions measured on ECG-triggered, balanced steady-state free precession (SSFP) MRA as alternative to CE-MRA.

**Methods:**

All patients, with varying medical conditions, referred for thoracic aortic examination between September 2016 and March 2018, who underwent non-ECG-triggered CE-MRA and SSFP-MRA (1.5 T) were retrospectively included (*n* = 30). Aortic dimensions were measured after double-oblique multiplanar reconstruction by two observers at nine landmarks predefined by literature guidelines. Image quality was scored at the sinus of Valsalva, mid-ascending aorta and mid-descending aorta by semi-automatically assessing the vessel sharpness.

**Results:**

Aortic dimensions showed high agreement between non-ECG-triggered CE-MRA and SSFP-MRA (*r* = 0.99, *p* < 0.05) without overestimation or underestimation of aortic dimensions in SSFP-MRA (mean difference, 0.1 mm; limits of agreement, − 1.9 mm and 1.9 mm). Intra- and inter-observer variabilities were significantly smaller with SSFP-MRA for the sinus of Valsalva and sinotubular junction. Image quality of the sinus of Valsalva was significantly better with SSFP-MRA, as fewer images were of impaired quality (3/30) than in CE-MRA (21/30). Reproducibility of dimensions was significantly better in images scored as good quality compared to impaired quality in both sequences.

**Conclusions:**

Thoracic aortic dimensions measured on SSFP-MRA and non-ECG-triggered CE-MRA were similar. As expected, SSFP-MRA showed better reproducibility close to the aortic root because of lesser motion artefacts, making it a feasible non-contrast imaging alternative.

**Key Points:**

• *SSFP-MRA provides similar dimensions as non-ECG-triggered CE-MRA.*

• *Intra- and inter-observer reproducibilities improve for the sinus of Valsalva and sinotubular junction with SSFP-MRA.*

• *ECG-triggered SSFP-MRA shows better image quality for landmarks close to the aortic root in the absence of cardiac motion.*

**Electronic supplementary material:**

The online version of this article (10.1007/s00330-020-06963-x) contains supplementary material, which is available to authorized users.

## Introduction

The incidence of aortic diseases is increasing with an ageing population [1]. In Western society, the incidence is rising further due to a growing group with overweight, hypertension and diabetes [2, 3]. Aortic disorders include dissection and aneurysms of the aorta, often in combination with aortic valve problems and atherosclerosis [4, 5]. Detailed imaging of the thoracic aorta with assessment of its dimensions is of major importance to monitor and detect aortic diseases in order to make valid clinical decisions [5, 6].

Computed tomography angiography is often used for clinical imaging of the thoracic aorta, because of speed and excellent spatial resolution [7]. However, it is less suitable for regular follow-up because it requires iodinated contrast and ionizing radiation [5, 7], making magnetic resonance angiography (MRA) a good alternative [5–8]. Contrast-enhanced (CE) MRA uses a gadolinium contrast bolus for 3-dimensional (3D) aortic depiction [5, 9]. Imaging of this contrast bolus requires a short acquisition time and does not allow for ECG triggering, resulting in movement artefacts close to the heart [10]. To avoid overestimation or underestimation of aortic diameters, it was therefore advised not to use ungated sequences in the sinus of Valsalva and sinotubular junction [9]. Furthermore, the possibility of gadolinium nephrotoxicity in patients with reduced renal function and the unknown effects of gadolinium deposition in the brain on later life caused the European Medicine Agency to advise minimisation of gadolinium contrast agent use [11–14]. Three-dimensional balanced steady-state free precession (SSFP) MRA of the thoracic aorta allows ECG triggering and navigator gating, and since it uses phase contrast, an additional contrast agent is not required [15]. Although ECG-triggered imaging was already proven to show superior image quality and similar aortic dimensions compared to CE-MRA [16–19], these sequences result in longer acquisition times and are therefore more prone to artefacts in patients with arrhythmias and clinically less-stable patients [20].

Measurements performed on either CE-MRA or SSFP-MRA must be interchangeable and reproducible so the best suited sequence can be chosen based on the patient’s current medical condition, without influencing follow-up decision making. A standardised measurement operating procedure is equally important, as differences in slice selection can cause (additional) variability [19]. Our objective of this study was to test the hypothesis that dimensions of thoracic aortic landmarks [5, 6] assessed with CE-MRA and SSFP-MRA are comparable and that intra- and inter-observer reproducibilities are improved on SSFP-MRA for landmarks close to the aortic root caused by better image quality.

## Materials and methods

### Study population

All patients referred for regular clinical examination of the thoracic aorta and who had both non-ECG-triggered CE-MRA and SSFP-MRA between September 2016 and March 2018 were identified (*n* = 37). Patients with aortic valve replacement or aortic stent placement were excluded, since this could interfere with aortic dimension assessment. Eventually 30 patients (19 males, mean age ± standard deviation: 41 ± 20 years) were retrospectively included in this single-centre study (Supplementary Table 1). This study was approved by the institutional review board with a waiver, and patients were excluded if an objection against use of their medical data for research was found in the institution’s objection registry.

### Image acquisition

Magnetic resonance imaging was performed on two 1.5-T systems (MAGNETOM Aera and MAGNETOM Avanto-fit, Siemens Healthineers) equipped with a phased-array, five-channel coil for cardiac imaging. SSFP-MRA was acquired using a 3D ECG- and navigator-gated, balanced SSFP-MRA sequence. The non-ECG-triggered CE-MRA was acquired with a T1-weighted sequence using the gadolinium-based contrast agent Dotarem [21]. Administered contrast volumes were 0.1 mmol/kg body weight at 2 mL/s. The order of sequences differed between patients, and subsequently, the acquisition of SSFP-MRA was contrast enhanced in 13 patients.

### Image analysis

**Fig. 1:**
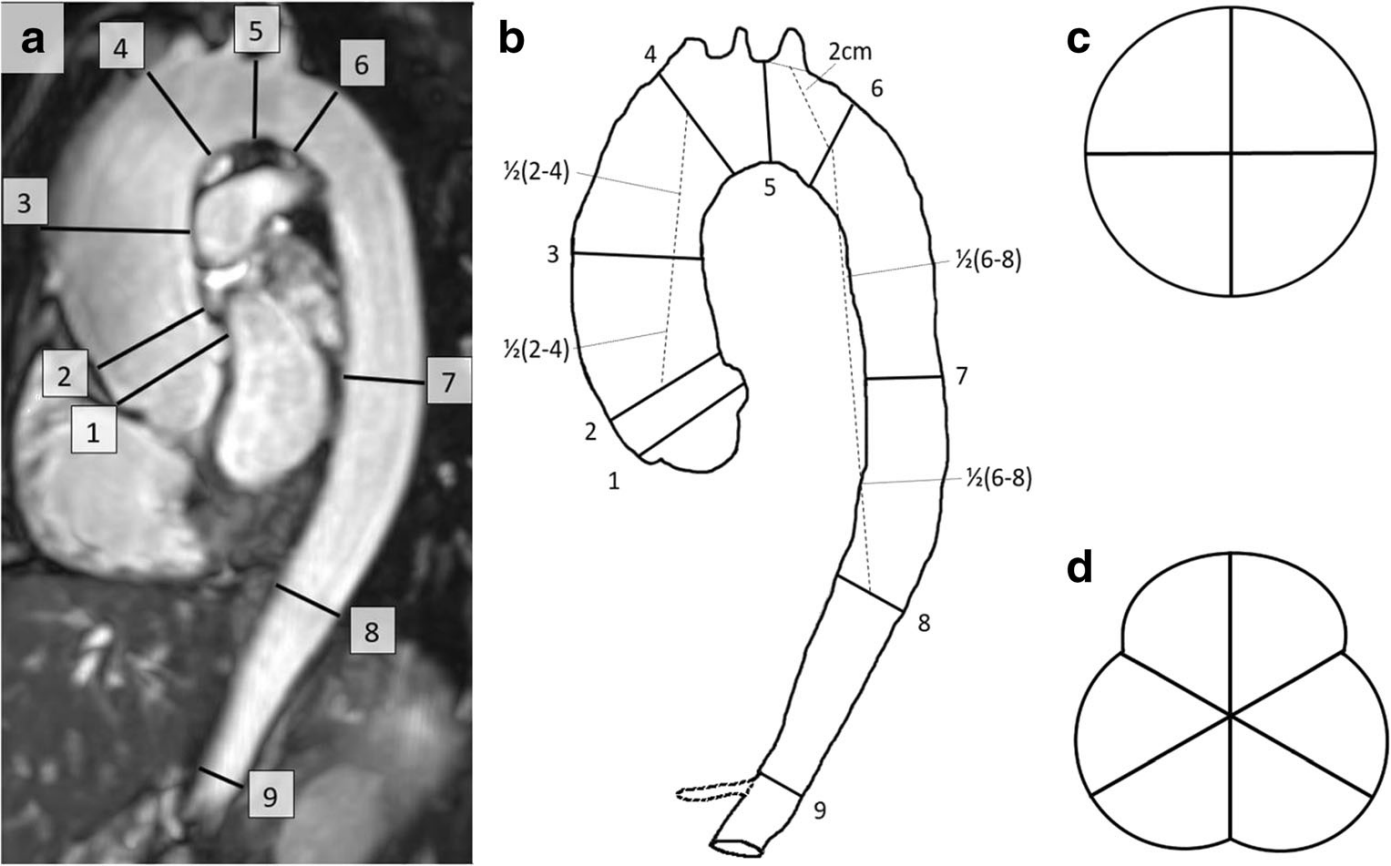
Overview of the thoracic landmarks. **a** The thoracic landmarks, including the abdominal aorta, on a SSFP-MRA as suggested by guidelines [5, 6]: (1) sinus of Valsalva, (2) sinotubular junction, (3) middle of the ascending aorta, (4) proximal aortic arch, (5) middle of the aortic arch, (6) proximal descending aorta, (7) middle of the descending aorta, (8) aorta at diaphragm and (9) abdominal aorta. **b** Schematic overview of the thoracic aorta on the SSFP-MRA with the origin of the celiac artery additionally traced. Landmarks 1, 2, 4, 5, 8 and 9 can be assessed using anatomical landmarks. Landmark 3 should be exactly in between landmarks 2 and 4; landmark 6 should be 2 cm distal from the centre of the lumen of the left subclavian artery; landmark 7 should be exactly in between landmarks 6 and 8. **c** Schematic anatomy of landmarks 2–9. **d** Schematic anatomy of the sinus of Valsalva. *SSFP* steady-state free precession; *MRA* magnetic resonance angiography

The acquired images were exported for post-processing to Circle cvi^42^ (Circle Cardiovascular Imaging) and anonymised in accordance with local Good Clinical Practice guidelines. The thoracic aorta was evaluated by two observers by measuring the aortic diameter at nine landmarks predefined by guidelines including the abdominal aorta [5, 6]: (1) sinus of Valsalva, (2) sinotubular junction, (3) middle of the ascending aorta (between landmarks 2 and 4), (4) proximal aortic arch (proximal to the brachiocephalic trunk), (5) middle of the aortic arch (between the left carotid and left subclavian arteries), (6) proximal descending aorta (2 cm distal to the left subclavian artery), (7) middle of the descending aorta (between landmarks 6 and 8), (8) aorta at diaphragm and (9) abdominal aorta (proximal to the celiac artery) (Fig. 1a).

The current guideline [5, 6] was adjusted with spacing rules for certain measurements without clear landmarks to ensure reproducible measurements with our standardised operating procedure. The landmarks sinus of Valsalva, sinotubular junction, proximal aortic arch, middle of the aortic arch, aorta at diaphragm and the abdominal aorta can be assessed without additional spacing rules, because of the clear anatomic landmarks. The remaining landmarks were established using a ruler to measure distances towards clear surrounding anatomy and placing a marker at the specific landmark (Fig. 1b).

Aortic dimensions were assessed using a multiplanar reformatting algorithm, enabling double-oblique measurements [9]. Lines were drawn in left–right (horizontal) and anterior–posterior direction (vertical) from inner edge to inner edge (Fig. 1c). In case of a visually oval-shaped aorta, the image was rotated, presenting the longest diameter in the left–right direction. The trefoil shape of the sinus of Valsalva allows for measuring the aortic diameters from cusp to cusp or from cusp to commissure. Since the post-processing guideline was inconclusive [9] and the literature was contradicting on the best method based on their reproducibility with echocardiographic diameters [22, 23], we opted to perform cusp-to-commissure measurements (Fig. 1d). Finally, the different lines within a landmark were averaged to the final aortic diameter. The first observer analysed all 30 scans twice for intra-observer reproducibility purposes, while the second observer analysed all scans once. Additionally, the second observer measured the maximum diameters of the ascending and descending aortas once in both CE-MRA and SSFP-MRA to test their similarity.

**Fig. 2:**
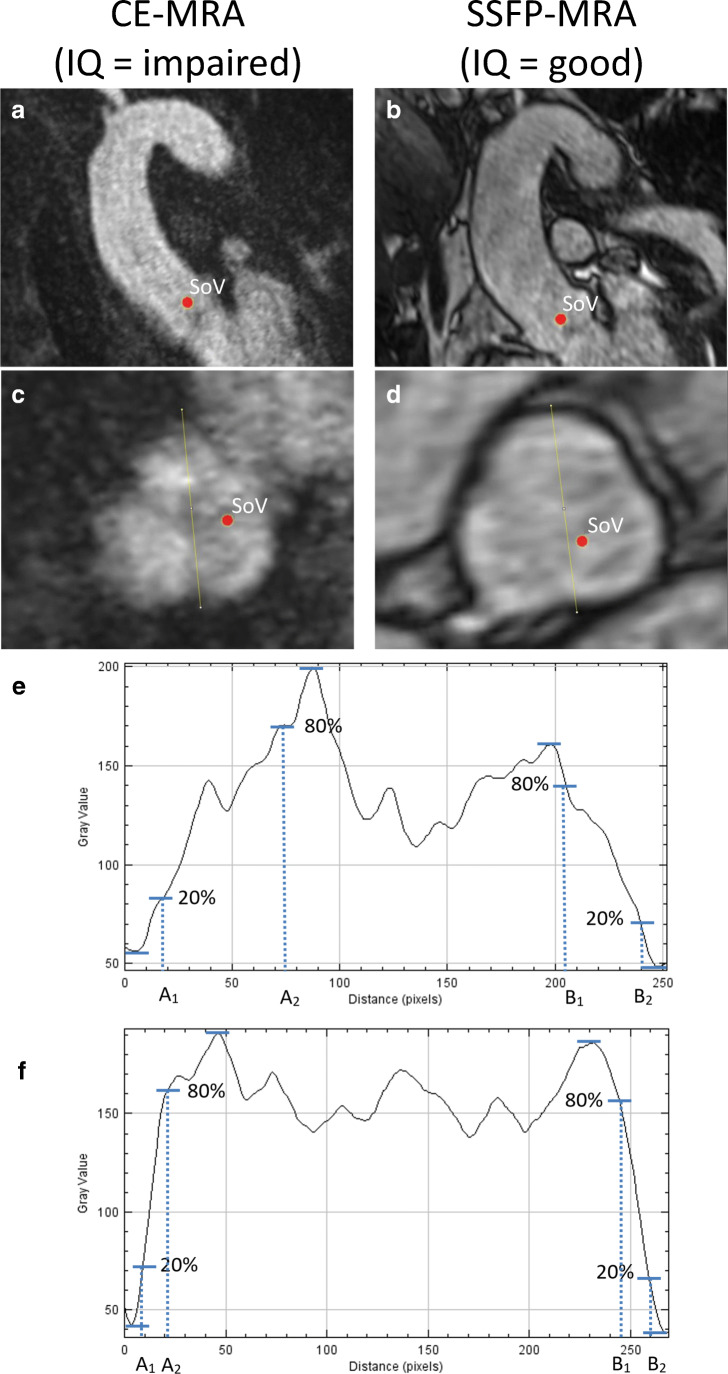

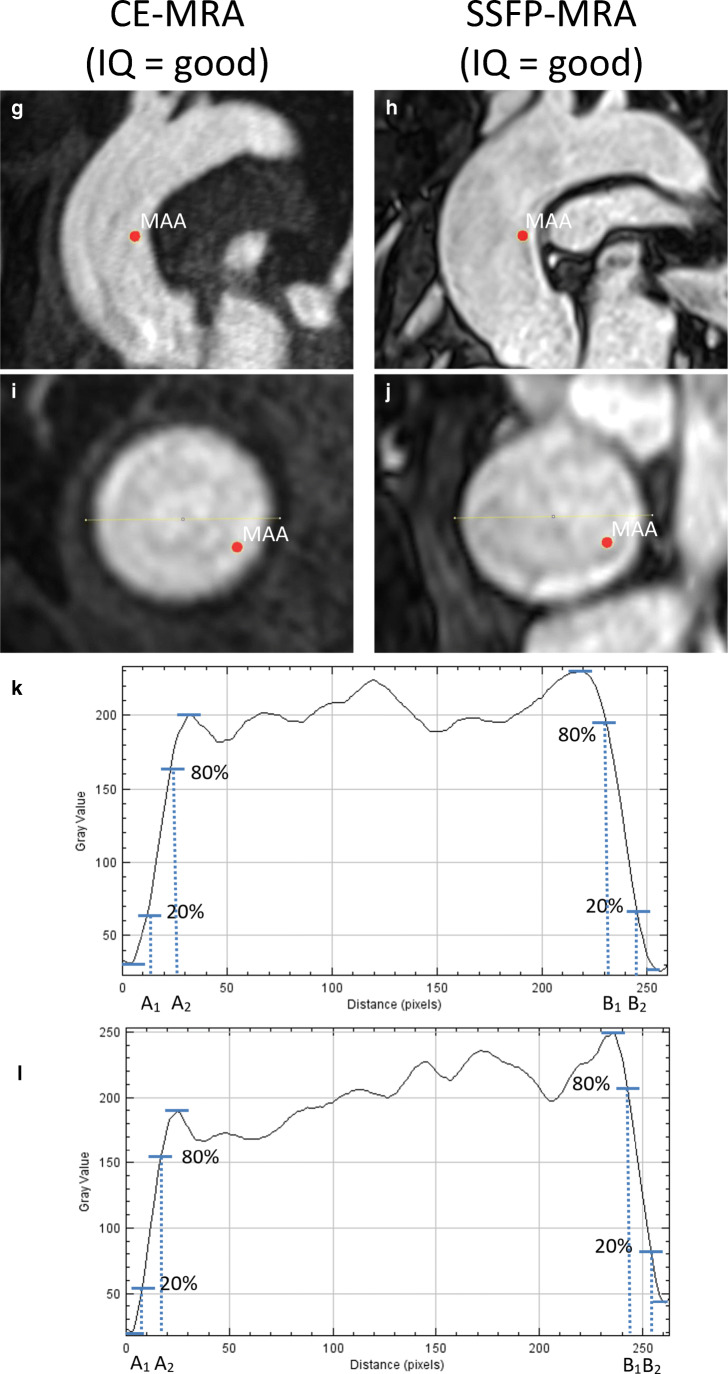
Image quality assessment. **a**, **b** The overview of the sinus of Valsalva (CE-MRA and SSFP-MRA, respectively). **g**, **h** The overview of the mid-ascending aorta (CE-MRA and SSFP-MRA, respectively). After multiplanar formation, panels **c** and **d** show the orthogonal overview of the sinus of Valsalva (CE-MRA and SSFP-MRA, respectively) and panels **i** and **j** show the orthogonal overview of the mid-ascending aorta (CE-MRA and SSFP-MRA, respectively). With the software ImageJ, three signal intensity profile plots were created, covering the sinus of Valsalva, and two plots in the mid-ascending aorta. Panels **e** and **k** and panels **f** and **l** show the profile plot of a single transecting line in CE-MRA and SSFP-MRA, respectively. For both sides of the profile plot, the maximum and minimum grey value was selected and its difference in grey value was calculated. Based on this difference, the *x*-axis values corresponding with relative grey values of 20% and 80% between the minimum and maximum grey values were selected (the area between these values is the transition zone). The number of pixels between the *x*-axis value corresponding with 20% (A_1_ for the left side, B_2_ for the right side) and 80% (A_2_ for the left side, B_1_ for the right side) on each side is a measure of vessel sharpness. The number of pixels of both transition zones on a single transecting line was added up and divided by the number of pixels between both transition zones, i.e. the distance between A_2_ and B_1_. The numbers of all transecting lines within a landmark were averaged, and its multiplicative inverse was the final sharpness score of that specific landmark. *CE* contrast-enhanced; *IQ* image quality; *MRA* magnetic resonance angiography; *SSFP* steady-state free precession

Accuracy in diameter measurements depends on the sharpness of transition between the vessel and the surrounding tissue. Therefore, the vessel sharpness was used to quantify the image quality and was measured at three landmarks—sinus of Valsalva, mid-ascending aorta and mid-descending aorta—to investigate the image quality along the thoracic aorta and the overall influence of image quality on reproducibility in CE-MRA and SSFP-MRA. In order to minimise observer bias, we semi-automatically calculated the vessel sharpness using the method of Groves et al [24]. The vessel sharpness was measured by creating signal intensity profile plots on the orthogonal overview of the aortic lumen with the software package ImageJ, version 1.52. For the sinus of Valsalva, this was performed using three transecting lines, and for the mid-ascending and mid-descending aortas, two transecting lines were used. The vessel sharpness was measured by determining the number of pixels between the pixels with 20% and 80% (transition zone) of the maximum signal intensity on each side of a transecting line (Fig. 2). Subsequently, the number of pixels of both transition zones of a transecting line was added up and divided by the number of pixels between both transition zones on that transecting line. Finally, the measurements of all transecting lines within a single landmark were averaged, and the multiplicative inverse of this number was the final sharpness score for that specific landmark [24]. A conversion table was proposed to classify the vessel sharpness score as an ordinal image quality score for analysis purposes. The scores were as follows: 0 (poor, non-diagnostic (vessel sharpness 0–2)), 1 (impaired image quality that may lead to misdiagnosis (vessel sharpness 2–3)) and 2 (good (vessel sharpness > 3)).

### Statistical analysis

Statistical analysis was performed using IBM SPSS Statistics for Windows, version 24 (IBM Corp.). *p* values < 0.05 were considered significant. The agreement between all paired CE-MRA and SSFP-MRA measurements was determined with the intraclass correlation coefficient (ICC) and Bland–Altman analysis [25]. For each individual landmark, the difference was calculated between CE-MRA and SSFP-MRA, and also, the ICC was determined [25]. Furthermore, the paired sample *t* test was used to check for systematic bias between dimensions of CE-MRA and SSFP-MRA. The intra- and inter-observer variabilities in both CE-MRA and SSFP-MRA were calculated for all landmarks combined and also separately for each landmark, and were reported as the mean difference and the 95% limits of agreement (LoA). The intra- and inter-observer variabilities of both MRA sequences were compared with a paired sample *t* test to test for better intra- and inter-observer reproducibilities with one of the sequences in one or more landmarks.

The mean image quality of both techniques was compared using the Wilcoxon rank test. Boxplots were created to visualise the influence of image quality on intra- and inter-observer variabilities. ANOVA tests were performed to check for significant differences in the reproducibility between the different image quality scores. Subsequently, post hoc tests were performed to investigate which image quality provided significantly improved reproducibility.

## Results

### Mean aortic dimensions in CE-MRA and SSFP-MRA

**Fig. 3:**
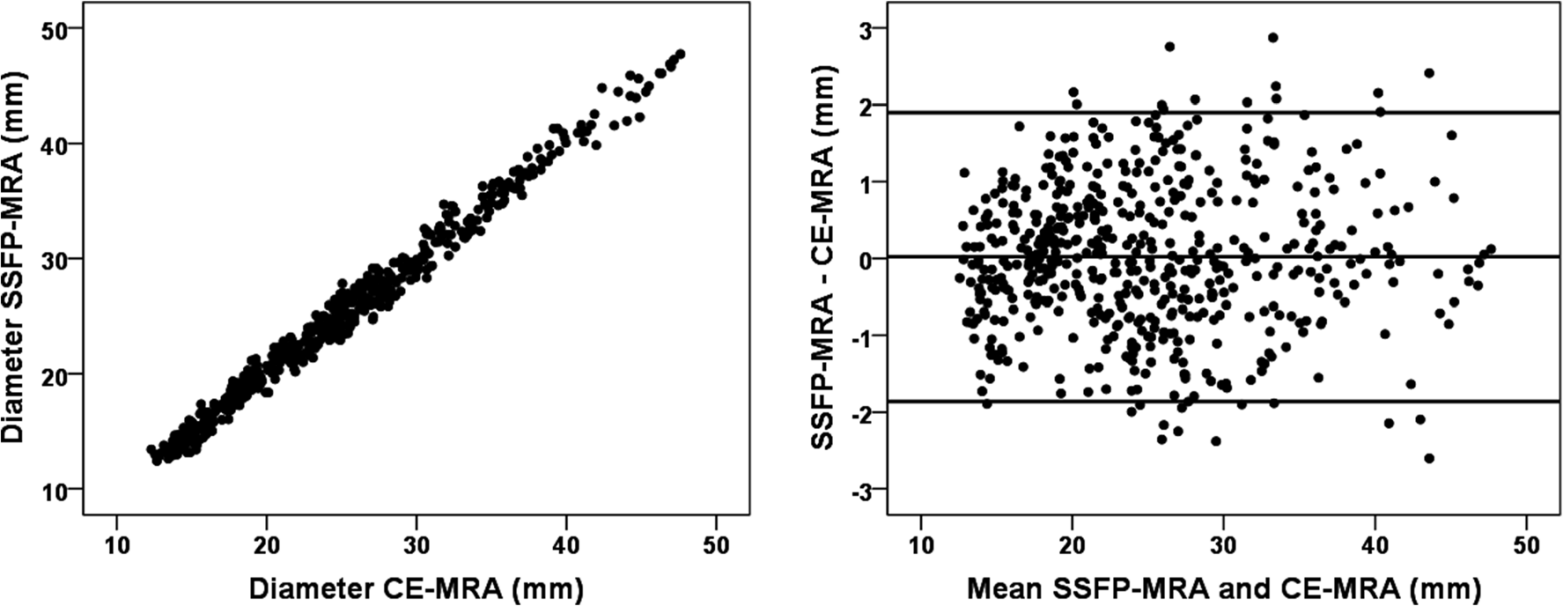
Bland–Altman analysis of all aortic dimensions of the predefined landmarks measured with both SSFP-MRA and CE-MRA. *CE* contrast-enhanced; *MRA* magnetic resonance angiography; *SSFP* steady-state free precession

All single measurements of all landmarks combined measured on non-ECG-triggered CE-MRA and SSFP-MRA showed high correlation (*r* = 0.99, *p* < 0.05) (Fig. 3). The Bland–Altman analysis showed no overestimation or underestimation of aortic dimensions with SSFP-MRA (mean difference, 0.1 mm; LoA, [− 1.9 mm;1.9 mm]) (Fig. 3). The mean diameters of all landmarks assessed using the standardised operating procedure with both CE-MRA and SSFP-MRA are provided in Supplementary Table 2. The correlation was excellent for all landmarks and least at the mid-aortic arch (*r* = 0.97, *p* < 0.05). The paired sample *t* test showed no systematic bias between the two imaging techniques for any landmark, and the variation between CE-MRA and SSFP-MRA decreased from the sinus of Valsalva (mean difference, − 0.1 mm; LoA, [− 2.4 mm;2.2 mm]) towards the abdominal aorta (mean difference, − 0.1 mm; LoA, [− 1.7 mm;1.6 mm]) (Supplementary Table 2).

High agreement was found between CE-MRA and SSFP-MRA for the maximum diameters of the ascending aorta (*r* = 0.99, *p* < 0.05) and descending aorta (*r* = 0.98, *p* < 0.05). The Bland–Altman analysis showed no significant differences between sequences for both the ascending aorta (mean difference, 0.1 mm; LoA, [− 1.9 mm;2.1 mm]) and descending aorta (mean difference, − 0.0 mm; LoA, [− 2.0 mm;2.0 mm]) (Supplementary Fig. 1).

### Observer variability

The paired sample *t* test showed smaller intra-observer variability in SSFP-MRA (mean difference, 0.0 mm; LoA, [− 0.7 mm;0.7 mm]) compared to CE-MRA (mean difference, 0.2 mm; LoA, [− 0.9 mm;1.2 mm]) when considering all landmarks at once (*p* < 0.05). Moreover, when analysing the landmarks separately, only for the sinus of Valsalva, sinotubular junction and mid-ascending aorta the intra-observer variability was significantly smaller in SSFP-MRA compared to CE-MRA (*p* < 0.05), while for all other landmarks, the intra-observer variability was similar (Table 1, Supplementary Table 3). The intra-observer variability was largest for the sinus of Valsalva for both SSFP-MRA (mean difference, − 0.1 mm; LoA, [− 0.9 mm;0.7 mm]) and CE-MRA (mean difference, 0.1 mm; LoA, [− 2.1 mm;2.2 mm]).

**Table 1:** Intra- and inter-observer variabilities of both CE-MRA and SSFP-MRA

Landmark	Intra-observer variability^†^		Inter-observer variability^†^	
	CE-MRA	SSFP-MRA	CE-MRA	SSFP-MRA
Sinus of Valsalva	0.1 [− 2.1;2.2]	− 0.1 [− 0.9;0.7]*	− 0.1 [− 2.4;2.2]	− 0.1 [− 1.4;1.1]*
Sinotubular junction	0.2 [− 0.8;1.1]	0.1 [− 0.6;0.7]*	0.1 [− 1.7;2.0]	− 0.2 [− 1.3;0.9]*
Mid-ascending aorta	0.4 [− 0.4;1.1]	0.0 [− 0.8;0.8]*	0.0 [− 1.2;1.3]	− 0.0 [− 1.2;1.1]
Proximal aortic arch	0.1 [− 0.5;0.7]	0.0 [− 0.6;0.7]	− 0.1 [− 1.0;0.9]	− 0.0 [− 0.8;0.7]
Mid-aortic arch	0.1 [− 0.6;0.8]	0.1 [− 0.6;0.8]	− 0.2 [− 1.6;1.2]	0.1 [− 1.1;1.3]
Proximal descending aorta	0.2 [− 0.8;1.2]	0.1 [− 0.6;0.8]	− 0.3 [− 1.2;0.6]	− 0.0 [− 1.1;1.0]
Mid-descending aorta	0.2 [− 0.4;0.9]	0.0 [− 0.8;0.8]	− 0.1 [− 1.2;1.0]	− 0.1 [− 1.0;0.9]
Aorta at diaphragm	0.1 [− 0.7;0.9]	− 0.1 [− 0.7;0.5]	− 0.1 [− 1.1;1.0]	− 0.2 [− 1.1;0.7]
Abdominal aorta	0.2 [− 0.4;0.7]	− 0.1 [− 0.8;0.6]	− 0.2 [− 1.1;0.8]	− 0.4 [− 1.5;0.8]

*CE* contrast-enhanced, *MRA* magnetic resonance angiography, *SSFP* steady-state free precession

^†^Variability presented as mean difference [limits of agreement] (mm)

*Significantly less variability in SSFP-MRA compared to CE-MRA as analysed with the paired sample *t* test (*p* < 0.05)

**Fig. 4:**
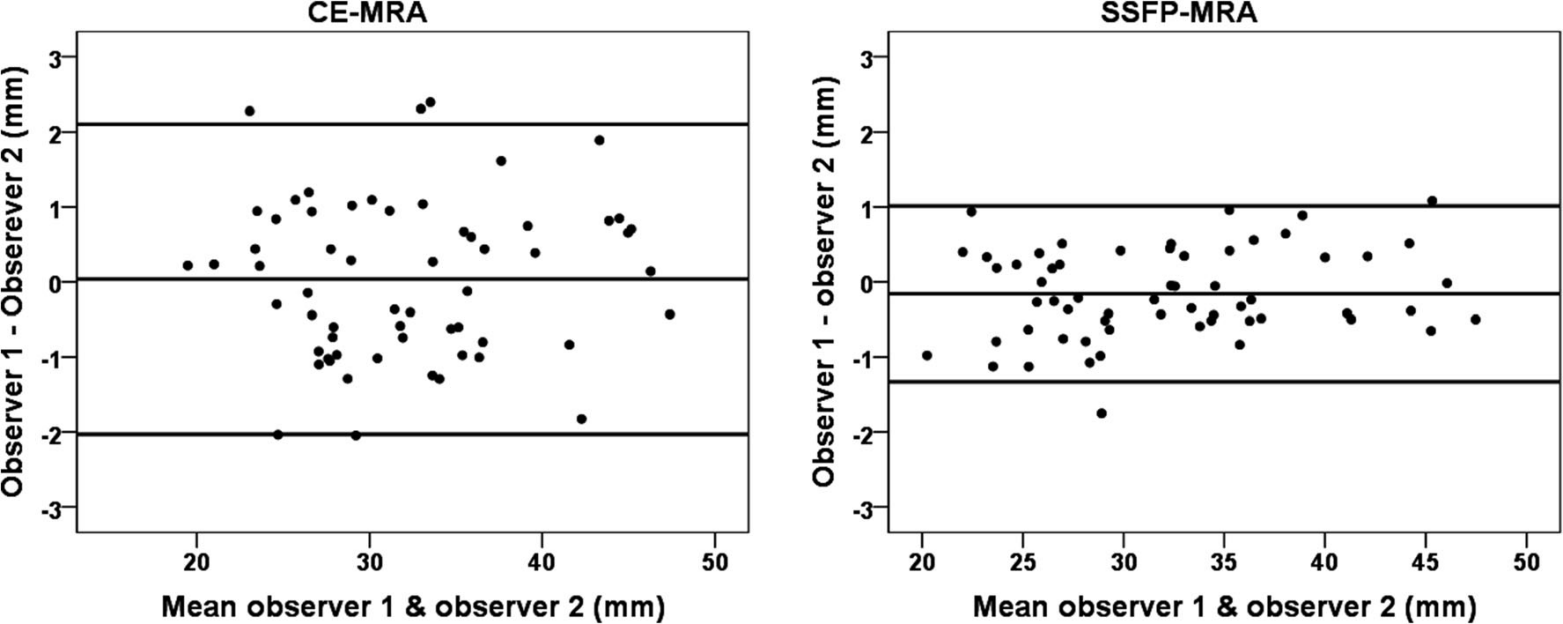
Bland–Altman analysis of the inter-observer variability of the sinus of Valsalva and sinotubular junction with CE-MRA (left) and SSFP-MRA (right). *CE* contrast-enhanced; *MRA* magnetic resonance angiography; *SSFP* steady-state free precession

Likewise, the paired sample *t* test between observers showed smaller inter-observer variability in SSFP-MRA (mean difference, − 0.1 mm; LoA, [− 1.2 mm;1.0 mm]) compared to CE-MRA (mean difference, − 0.1 mm; LoA, [− 1.5 mm;1.3 mm]) when all landmarks were included (*p* < 0.05). After analysing all landmarks separately, the inter-observer variability was significantly smaller with SSFP-MRA for the sinus of Valsalva and the sinotubular junction (*p* < 0.05) (Fig. 4, Table 1, Supplementary Table 3). Similarly, the inter-observer variability was the largest for the sinus of Valsalva for both SSFP-MRA (mean difference, − 0.1 mm; LoA, [− 1.4 mm;1.1 mm]) and CE-MRA (mean difference, 0.1 mm; LoA, [− 2.4 mm;2.2 mm]).

### Image quality

**Fig. 5:**
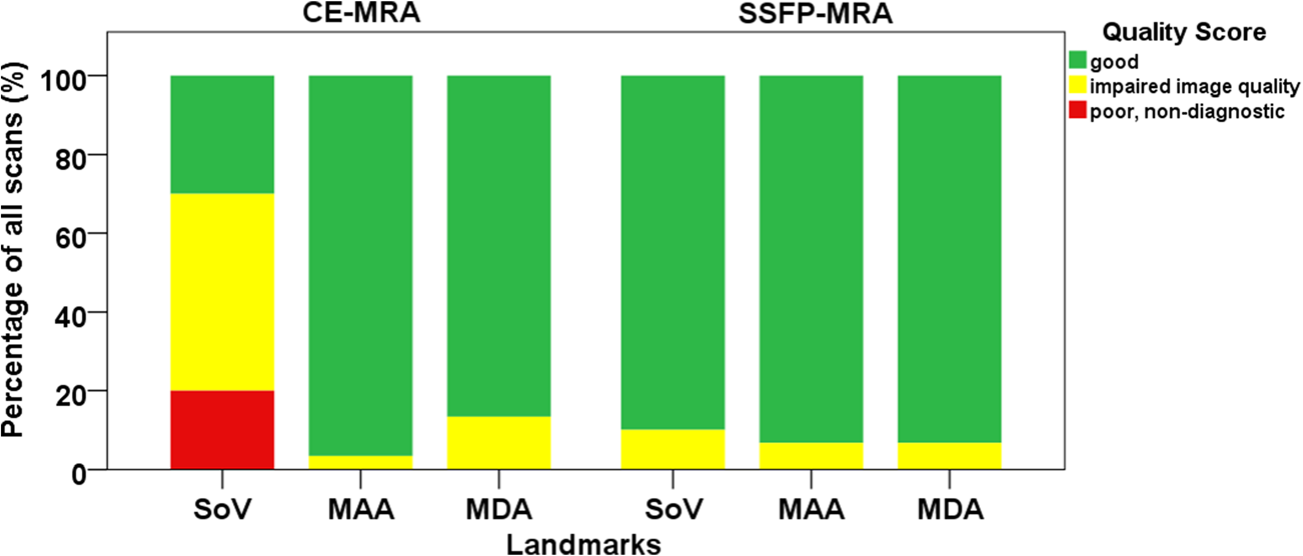
Overview of the image quality. *CE* contrast-enhanced; *MRA* magnetic resonance angiography; *SSFP* steady-state free precession; *SoV* sinus of Valsalva; *MAA* middle of the ascending aorta; *MDA* middle of the descending aorta

The overview of image quality results is presented in the stacked bar chart (Fig. 5). For the sinus of Valsalva, 70% of CE-MRA showed impaired quality or worse (score 1 or 0), while this was 10% with SSFP-MRA. Consequently, the average image quality of the sinus of Valsalva was significantly better with SSFP-MRA than with CE-MRA (1.9 ± 0.3 vs 1.1 ± 0.7, *p* < 0.05). The image quality was similar between CE-MRA and SSFP-MRA for the mid-ascending aorta (2.0 ± 0.2 vs 1.9 ± 0.3, respectively, *p* = 0.56) and for the mid-descending aorta (1.9 ± 0.3 vs 1.9 ± 0.3, respectively, *p* = 0.41). None of the images of the mid-ascending or mid-descending aorta was quantified as non-diagnostic in either CE-MRA or SSFP-MRA.

### Image quality on observer variability

**Fig. 6:**
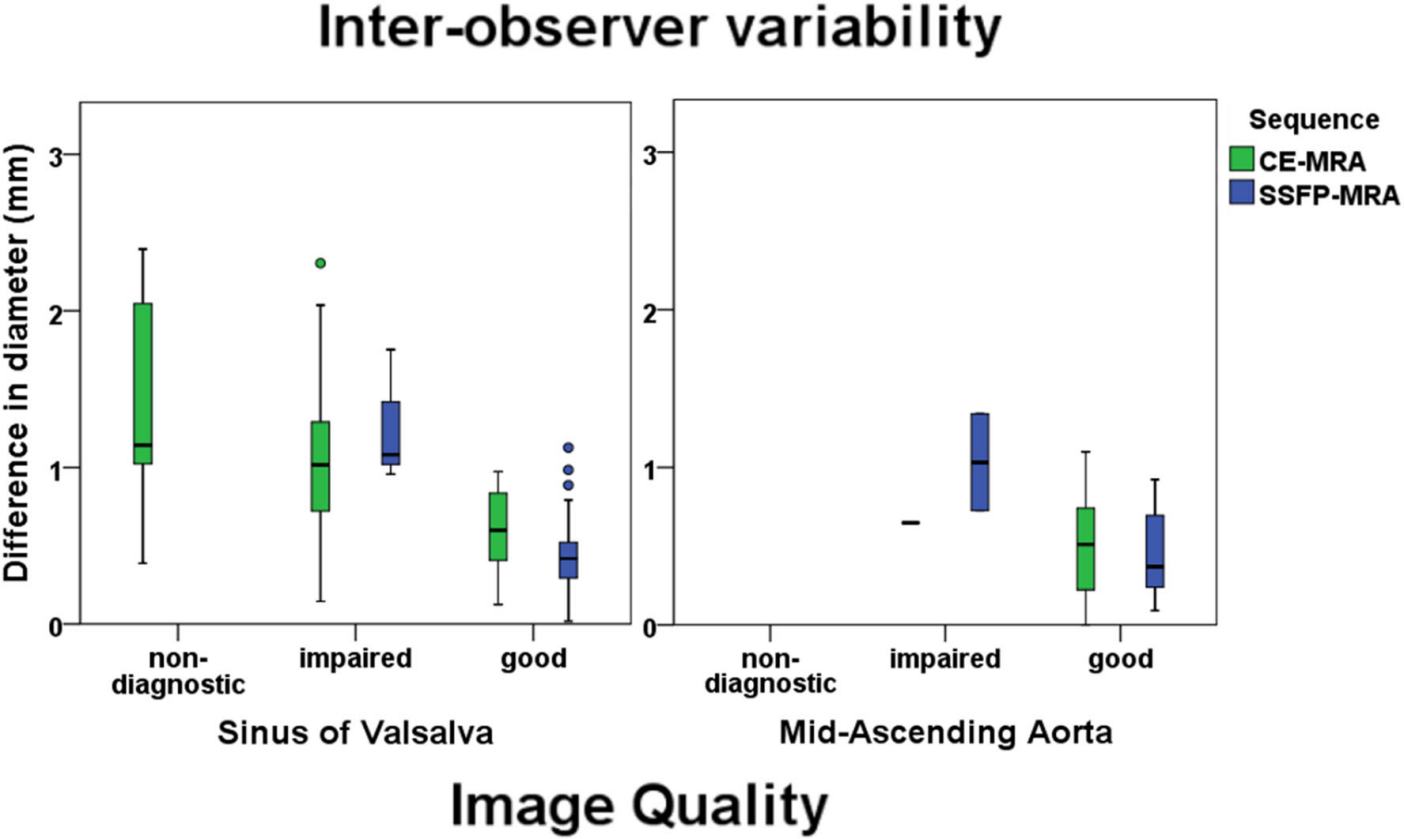
Boxplot of the inter-observer variability for the different image quality scores. On the *x*-axis, the image quality score of both CE-MRA and SSFP-MRA is presented for the landmarks sinus of Valsalva (left) and the mid-ascending aorta (right). The *y*-axis shows the variability in mm of the inter-observer measurements corresponding with the different image quality scores. *CE* contrast-enhanced; *MRA* magnetic resonance angiography; *SSFP* steady-state free precession

The inter-observer variability of the sinus of Valsalva and the mid-ascending aorta for all different image quality scores is visualised separately per sequence in a boxplot (Fig. 6). Boxplots for the mid-descending aorta and the intra-observer variability are provided in Supplementary Fig. 2. The sinus of Valsalva showed smaller inter-observer variability for images scored as good (score 2) compared to images scored as impaired image quality or worse (score 1 and 0) in both sequences (Fig. 6). The small number of scans scored as impaired quality in the mid-ascending aorta also suggested less variability for images with good image quality (Fig. 6).

The ANOVA tests were significant for both intra- and inter-observer variabilities of CE-MRA and SSFP-MRA, indicating that the degree of intra- and inter-observer variabilities is influenced by image quality. Post hoc tests showed that the variability of landmarks scored as non-diagnostic or impaired in CE-MRA were significantly larger than landmarks scored as good in CE-MRA. The images scored as impaired quality within SSFP-MRA also showed more variability than images scored as good. There was no difference in variability between CE-MRA and SSFP-MRA for the same image quality scores; e.g. variability in CE-MRA with good image quality was similar to SSFP-MRA with good image quality.

## Discussion

In this study, we compared non-ECG-triggered CE-MRA and SSFP-MRA to investigate their interchangeability and reproducibility in aortic dimension assessment. Aortic dimensions were assessed at nine predefined landmarks using a standardised protocol adjusted with spacing rules for landmarks without clear surrounding anatomy to accurately measure aortic dimensions, resulting in similar dimensions between CE-MRA and SSFP-MRA (Fig. 3, Supplementary Table 2).

The high correlation of aortic dimensions between non-ECG-triggered CE-MRA and SSFP-MRA was in agreement with previous studies focusing on thoracic aortic dimensions [19, 26–28]. Aortic dimensions were not overestimated or underestimated with SSFP-MRA compared to CE-MRA in studies reporting dimensions assessed on a cross-sectional overview [15, 19, 26, 28]. Conversely, Veldhoen et al [27] reported underestimation of dimensions with SSFP-MRA. They, however, acquired 2D instead of 3D images and analysed aortic dimensions on the para-sagittal plane, leading to perhaps less accurate dimensions. The Society of Cardiovascular Magnetic Resonance recommends aortic dimension assessment on double-oblique multiplanar images, requiring 3D sequences [9]. In our study, the difference between CE-MRA and SSFP-MRA tended to decrease for landmarks more distal from the sinus of Valsalva. This finding was confirmed by Von Knobelsdorff-Brenkenhoff et al [19], the only other study reporting differences between 3D SSFP-MRA and CE-MRA for multiple landmarks along the thoracic aorta. However, in their study, the mid-ascending aorta showed relatively high variability compared to other aortic landmarks. As their study population primarily consisted of patients with suspicion or control of ascending aortic aneurysms (74%), they suggested that inaccurate slice selection resulted in large differences of aortic diameters within aortic aneurysms, emphasizing the importance of accurate slice selection. In our study, spacing rules were used for locations without clear landmarks, which possibly explains the lower variability. Moreover, as few of our included subjects were suffering from an ascending aortic aneurysm, less dimension variability can be expected.

The intra- and inter-observer variabilities of the sinus of Valsalva and sinotubular junction were significantly smaller in SSFP-MRA than in non-ECG-triggered CE-MRA (Table 1) which is in accordance with previous studies [29–31] as is the similar variability of landmarks distal from the aortic root [22, 26, 29, 30, 32]. Only Von Knobelsdorff-Brenkenhoff et al [19] reported similar intra- and inter-observer variabilities for all landmarks, suggesting no reproducibility improvement of the aortic root measurements with SSFP-MRA.

Landmarks close to the aortic root are sensitive to cardiac motion in untriggered sequences [10]. We assessed the image quality on three landmarks along the aorta to study differences in image quality. The image quality of the sinus of Valsalva was significantly better with SSFP-MRA (*p* < 0.05), while the image quality of the mid-ascending and mid-descending aortas was similar with non-ECG-triggered CE-MRA (Fig. 6). Most previous studies assessed the image quality of the thoracic aorta with ECG-triggered SSFP-MRA versus non-ECG-triggered CE-MRA using visual classification criteria [18, 19, 28, 29, 31, 33], whereas one lacked these criteria [15] and another two studies used a semi-automatic approach [24, 30]. Despite the variation of methods, all studies reported improved image quality with SSFP-MRA for the aortic root [15, 19, 28–31, 33, 34]. Some studies also reported increased image quality of the mid-ascending aorta [19, 24, 29, 30, 34] and mid-descending aorta [19, 29]. These improvements were small compared to improvements in the aortic root, probably caused by similarly good average image quality of CE-MRA for these landmarks.

Only a limited number of the studies mentioned above reported results of both observer reproducibility and image quality in SSFP-MRA and non-ECG-triggered CE-MRA [19, 29–31]. Potthast et al [30] demonstrated superior image quality and reproducibility with SSFP-MRA for the sinus of Valsalva, sinotubular junction and ascending aorta. Bannas et al [31] also reported significantly improved image quality and reproducibility with SSFP-MRA compared to CE-MRA for the sinus of Valsalva and sinotubular junction; however, the reproducibility of other landmarks was not reported. Furthermore, van Kesteren et al [29] reported the lowest and highest image qualities with CE-MRA, with corresponding lowest and highest inter-observer agreements in measuring diameters, in the sinus of Valsalva and the distal ascending aorta, respectively. Although Von Knobelsdorff-Brenkenhoff et al [19] showed improved image quality for all landmarks with SSFP-MRA compared to CE-MRA along the aorta, the overall intra- and inter-observer reproducibilities were similar between both sequences, suggesting that image quality does not influence observer reproducibility. To our knowledge, our study is the first to directly link the reproducibility of aortic dimensions with image quality, since previous studies only reported values of reproducibility and image quality for the entire study population. We demonstrated decreased reproducibility of dimensions in images with impaired quality which is in accordance with the main literature findings, suggesting that impaired CE-MRA quality at the aortic root causes impaired reproducibility.

Surgery indications for thoracic aortic aneurysms are mainly based on either absolute aortic diameters or size increase over 1 year [5]. The lower threshold for aneurysms is stated in guidelines and depends on the aortic segment of the dilation—ascending aorta, aortic arch or descending aorta—and on the medical condition of the patient. The size increase of > 3 mm/year should be measured using repetitive measurements obtained with the same imaging technique on the exactly the same aortic level, and this increase should be checked using an alternative technique to test its consistency when it impacts the therapeutic decision [5]. To prevent overestimation or underestimation of aortic size increase between repetitive measurements when using both non-ECG-triggered CE-MRA and SSFP-MRA in follow-up, similarity of dimensions is required which was demonstrated in this study. However, the variability in and between observers within the same sequence will inevitably add some uncertainty about the actual size increase, which is especially the case in measuring the aortic root in CE-MRA and is caused by motion artefacts. When aortic root dilatation is suspected, ideally SSFP-MRA should be performed to minimise both false negative and positive surgery indications. The use of CE-MRA or SSFP-MRA does not influence the accuracy of the assessed aortic size increase in suspected aortic dilation in other segments, since variation was found to be similar between sequences. To ensure the selection of the exactly the same aortic level in follow-up measurement, it may be helpful to measure the distances from that aortic level towards the closest proximal and distal anatomic landmark and add this information to the medical report.

This study has some limitations. In 13 of the 30 subjects, the SSFP-MRA was performed after gadolinium administration where it ideally should have been performed prior to gadolinium administration to prevent image quality bias from contrast agents. Nevertheless, there was no measurable image quality and reproducibility improvement in the SSFP-MRA acquired after compared to before gadolinium administration (*p* = 0.45 and *p* = 0.29, respectively). Furthermore, the second observer performed the analysis once and therefore the intra-observer variability was based on the reproducibility measurements of the first observer.

The aortic dimensions of predefined locations were similar on SSFP-MRA and non-ECG-triggered CE-MRA using a standardised operating procedure to ensure reproducible slice selection. SSFP-MRA showed improved reproducibility for landmarks close to the aortic root, since these landmarks in non-ECG-triggered CE-MRA are sensitive to cardiac motion and therefore result in impaired image quality. Contrast-free SSFP-MRA seems to be a good alternative for assessment of thoracic aortic dimensions with improved image quality and reproducibility.

## Electronic supplementary material

ESM 1 (DOCX 162 kb)

## Abbreviations

3D: 3-dimensional
CE: Contrast-enhanced
ICC: Intraclass correlation coefficient
LoA: Limits of agreement
MRA: Magnetic resonance angiography
SSFP: Steady-state free precession
