# Supplementary material for: Validation of thoracic aortic dimensions on ECG-triggered SSFP as alternative to contrast-enhanced MRA
Source: Eur Radiol. 2020 Jun 7;30(11):5794–804. doi: 10.1007/s00330-020-06963-x (PMC7554008; doi:10.1007/s00330-020-06963-x)
Supplement: Supplementary file 1 — (DOCX 162 kb) [file 330_2020_6963_MOESM1_ESM.docx]

Supplementary Table 1 Study population demographics

| **Demographics** | **Results** |
| --- | --- |
| Number of patients | 30 |
| Age (years) | 41 ± 20 |
| Gender, male | 19 (63%) |
| Weight (kg) | 75 ± 20 |
| Indication for MRA |  |
| - Follow-up aortic dilatation | 15 (50%) |
| - Turner syndrome | 4 (13%) |
| - Follow-up coarctation | 2 (7%) |
| - Syndrome of Marfan | 1 (3%) |
| - Other | 8 (27%) |

**Data was expressed as mean ± standard deviation or as number (percentage).**

Supplementary Table 2 Comparison of aortic diameters

|  | **Diameter** |  |  | **Difference** |  | **ICC** |
| --- | --- | --- | --- | --- | --- | --- |
| **Landmark** | **CE-MRA^†^** | **SSFP-MRA^†^** |  | **Mean [LoA]*** |  | **r** |
| Sinus of Valsalva | 34.2 ± 6.7 | 34.0 ± 6.8 |  | – 0.1 [-2.4; 2.2] |  | 0.99 |
| Sinotubular junction | 30.3 ± 6.5 | 30.3 ± 6.5 |  | 0.0 [-2.0; 2.0] |  | 0.99 |
| Mid-ascending aorta | 33.0 ± 6.2 | 33.1 ± 6.3 |  | 0.2 [-1.8; 2.2] |  | 0.99 |
| Proximal aortic arch | 27.5 ± 5.4 | 27.3 ± 5.5 |  | – 0.2 [-2.1; 2.7] |  | 0.98 |
| Mid-aortic arch | 21.5 ± 4.4 | 21.6 ± 4.5 |  | 0.1 [-2.1; 2.2] |  | 0.97 |
| Proximal desc. aorta | 20.1 ± 4.7 | 20.1 ± 4.7 |  | 0.0 [-1.6; 1.6] |  | 0.99 |
| Mid-descending aorta | 20.4 ± 4.1 | 20.6 ± 4.3 |  | 0.2 [-1.6; 1.9] |  | 0.98 |
| Aorta at diaphragm | 19.1 ± 4.0 | 19.3 ± 4.1 |  | 0.1 [-1.3; 1.5] |  | 0.98 |
| Abdominal aorta | 18.2 ± 3.7 | 18.1 ± 3.8 |  | -0.1 [-1.7; 1.6] |  | 0.98 |

**Mean diameter per landmark for both sequences, the difference and the intraclass correlation coefficient.**

**^†^Data presented as mean ± standard deviation (mm)**

**^*^Difference presented as mean difference [limits of agreement] (mm)**

***CE* contrast-enhanced; *LoA* limits of agreement; *MRA* magnetic resonance angiography; *SSFP* steady-state free precession; *ICC* intraclass correlation coefficient.**

Supplementary Table 3 Intra- and inter-observer variability

**^†^Data presented as the range of variability (mm) between reproducibility measurements and the maximum disagreement percentage (%).**

***CE* contrast-enhanced; *MRA* magnetic resonance angiography; *SSFP* steady state free precession**

| **Landmark** | **Intra-observer variability^†^** | |  | **Inter-observer variability^†^** | |
| --- | --- | --- | --- | --- | --- |
|  | **CE-MRA** | **SSFP-MRA** |  | **CE-MRA** | **SSFP-MRA** |
| Sinus of Valsalva | – 1.9 – 2.3; 6% | – 1.1 – 1.0; 3% |  | – 2.1 – 2.4; 8% | – 1.8 – 1.1; 6% |
| Sinotubular junction | – 1.4 – 0.8; 5% | – 0.5 – 0.9; 4% |  | – 1.8 – 2.3; 10% | – 1.1 – 0.9; 5% |
| Mid-ascending aorta | – 0.4 – 1.3; 5% | – 0.7 – 1.3; 6% |  | – 1.1 – 1.0; 5% | – 0.8 – 1.3; 5% |
| Proximal aortic arch | – 0.6 – 0.7; 3% | – 0.4 – 1.2; 4% |  | – 0.9 – 0.9; 4% | – 0.8 – 0.6; 3% |
| Mid-aortic arch | – 0.8 – 0.9; 4% | – 0.5 – 1.4; 5% |  | – 1.9 – 0.9; 10% | – 1.2 – 1.3; 6% |
| Proximal desc. aorta | – 1.4 – 1.1; 6% | – 0.6 – 0.6; 4% |  | – 1.3 – 0.7; 7% | – 1.3 – 1.1; 6% |
| Mid-desc. aorta | – 0.4 – 1.2; 9% | – 0.7 – 1.1; 6% |  | – 1.4 – 1.0; 9% | – 1.4 – 0.6; 6% |
| Aorta at diaphragm | – 0.4 – 1.2; 8% | – 0.5 – 0.8; 4% |  | – 1.2 – 1.4; 9% | – 1.1 – 0.9; 6% |
| Abdominal aorta | – 0.5 – 0.9; 7% | – 0.9 – 0.6; 5% |  | – 0.9 – 0.7; 7% | – 1.6 – 1.3; 8% |

**
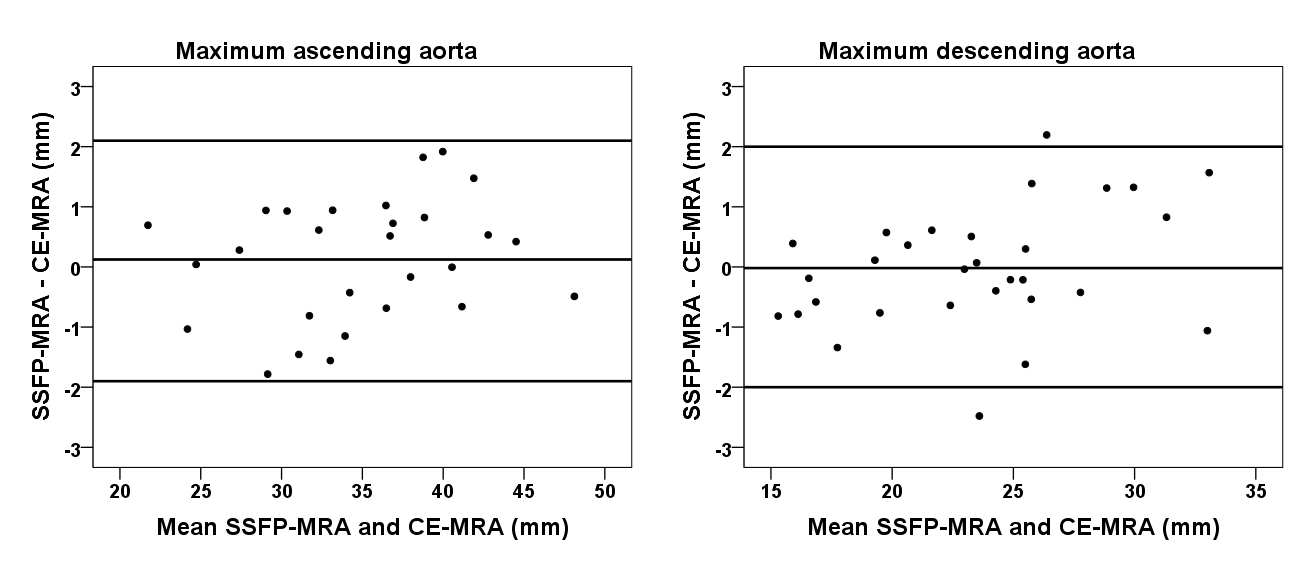
**

**Supplementary Fig. 1 Bland-Altman analysis of the differences between CE-MRA and SSFP-MRA for maximum diameter assessment of the ascending aorta (left) and descending aorta (right).**

***CE* contrast-enhanced; *MRA* magnetic resonance angiography; *SSFP* steady-state free precession**

**
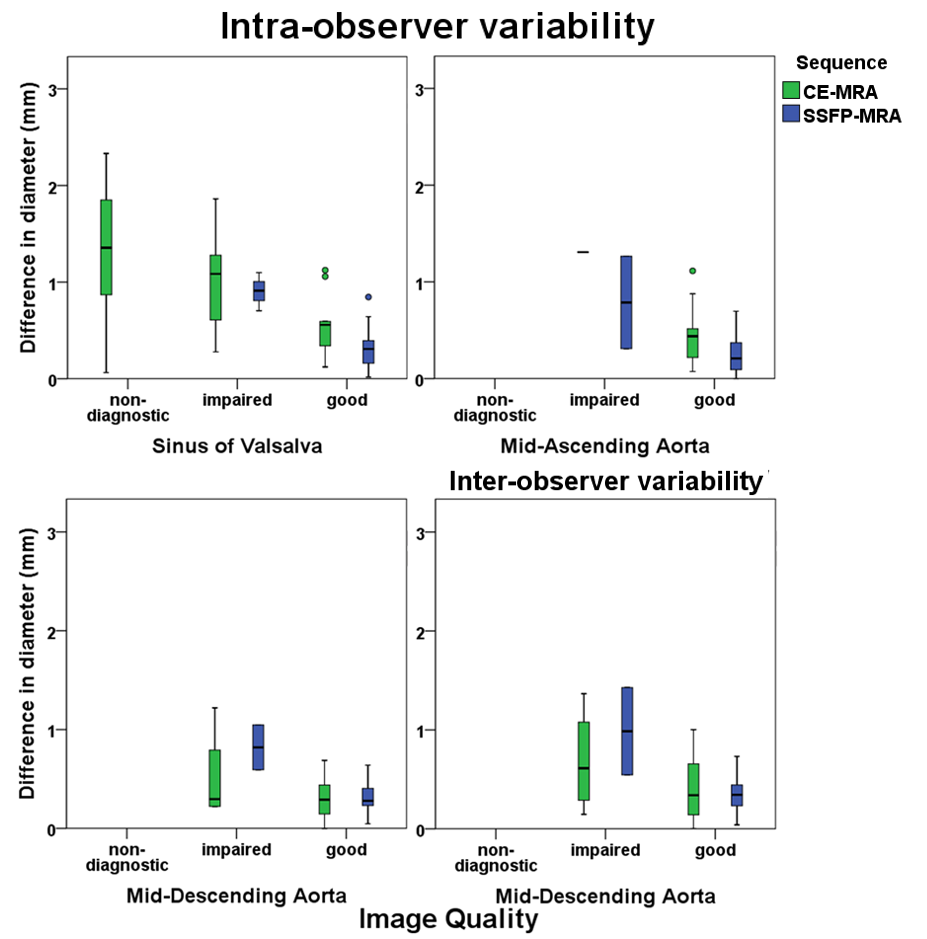
**

**Supplementary Fig. 2 Boxplot of the observer variability for the different image quality scores. The four panels represent the intra-observer variability in the sinus of Valsalva (top left), the mid-ascending aorta (top right), the mid-descending aorta (bottom left) and the inter-observer variability in the mid-descending aorta (bottom right). On the x-axis, the image quality score of both CE-MRA and SSFP-MRA is presented. The y-axis shows the variability in mm of the observer measurements corresponding with the different image quality scores.**

***CE* contrast-enhanced; *MRA* magnetic resonance angiography; *SSFP* steady state free precession**
